# Accumulation of heavy metals in mosses: a biomonitoring study

**DOI:** 10.1186/s40064-016-2524-7

**Published:** 2016-06-14

**Authors:** G. Macedo-Miranda, P. Avila-Pérez, P. Gil-Vargas, G. Zarazúa, J. C. Sánchez-Meza, C. Zepeda-Gómez, S. Tejeda

**Affiliations:** Laboratorio de Investigación en Ingeniería Ambiental, Instituto Tecnológico de Toluca, Av. Tecnológico S/N, Colonia Ex-Rancho La Virgen, 52140 Metepec, Estado de México Mexico; Instituto Nacional de Investigaciones Nucleares, Dirección de Investigación Tecnológica, Apartado Postal 18-1027, 11801 México, D.F. Mexico; Facultad de Química, Universidad Autónoma del Estado de México, Instituto Literario 100, 50000 Toluca, Estado De México Mexico; Facultad de Ciencias, Universidad Autónoma del Estado de México, Instituto Literario 100, 50000 Toluca, Estado De México Mexico

**Keywords:** Heavy metals, Atomic absorption spectrometry, Mosses, Biomonitoring, Atmospheric pollution

## Abstract

The metropolitan area of the Toluca Valley (MATV) extends over an area of 1208.55 km^2^ and has 1,361,500 inhabitants making it the fifth highest populated area in the country and the second highest in the state. The MATV has several environmental problems, with regards to the air quality. Particles PM_10_ and PM_2.5_ are considered to be the main pollutant due to these particles frequently exceeding the limit laid down in the standards of the air quality in the country. For this reason, samples of the mosses *Fabriona ciliaris and Leskea angustata* were collected at different sites in MATV, Mexico in order to establish the atmospheric deposition of heavy metals by means of the analysis of the mosses tissues. Results show the average metal concentrations in the mosses in the order of: Zn > Pb > Cr > Cd. The concentration capacities of heavy metals were higher in *Fabriona ciliaris* than *Leskea angustata.* Enrichment factors for Cr, Zn, Pb and Cd were obtained using the soils from the same sampling area. Enrichment factors results show that Cr is conservative in both sampling seasons with a terrigenous origin; Zn is moderately enriched in both sampling seasons and mainly associated to pedological-soil or substrate contribution and anthropogenic activities and Cd is highly enriched in the rainy season and Pb is highly enriched in both sampling seasons, with a predominantly anthropogenic origin. This study provides information to be considered in the strategies for similar environmental problems in the world.

## Background

The MATV Mexico extends over an area of 1208.55 km^2^ with 1,361,500 inhabitants, for this reason, it has the second highest population in importance in the State of México and the fifth in the country. The MATV has several environmental problems, such as: changes in the land uses which have reduced agricultural and forest frontier, invasion of protected natural areas, deforestation, soil erosion, forest fires, burning of waste, as well as the emission of pollutants from industries and motor vehicles. With regard to the problem of the air quality, particles PM_10_ and PM_2.5_ are the most common pollutants because these particles frequently exceed the limits established in the Mexican standards of air quality (SMA-GEM 2007a; SMA-GEM 2012). Industrial activity and automotive traffic are strongly involved in the formation of particulate matter; as a result, the concentration of particles in urban areas is high compared to non-urban areas. Additionally, the particulate matter usually settles in areas far away from their origin (Machado et al. 2008).

In Mexico mosses are an important part of the urban landscape. 87 species and varieties of mosses of the Mexico City urban areas have been recorded, including several epiphyte species that have been removed. It is suggested that these changes in diversity are mainly due to the disappearance of natural habitats in the urban environment and air pollution (Delgadillo and Cárdenas 2000). It has been estimated that in the Toluca Valley the bryophyte consists of 136 species of mosses, both tropical and alpine affinity (Delgadillo 2009).

Mosses can be a useful tool for prospective studies that determine the conditions and characteristics of the environment. Mosses are ideal to evaluate pollution both in the field and laboratory studies, because of their diversity of habitats, structural simplicity and rapid multiplication rate. There are a large number of species of mosses that have been used to determine the presence of pollutants including heavy metals and radioactive materials (Galuszka 2006). Mosses can accumulate metals above their physiological needs due to the absence of cuticle in their tissues and the abundance of sites with exchangeable cations in their cell wall (Tessier and Boisvert 1999). Mosses are most commonly used to determine the atmospheric deposition of heavy metals, because they absorb elements principally from the atmosphere (Fernández and Carballeira 2001).

Mosses have several advantages as a bioindicator of heavy metal pollution: Mosses species have a wide distribution, they grow in urban, industrial and unpolluted areas, they uptake nutrients from precipitation, accumulate nutrients and pollutants by passive transport and metal concentrations in the moss’s tissues reflects the atmospheric deposition (Grodzinska and Szarek-Lukaszewska 2001).

The use of mosses for mapping the extension and direction of metal pollution from localized atmospheric sources is one of the most prominent uses of mosses in pollution monitoring. In most of these studies, the highest concentrations of the metals in the naturally–occurring mosses are observed in the immediate vicinities of the sources, and decreases with distance from the origin until background levels are reached (Onianwa 2001).

Several researches reported the use of different moss species as bioindicators for atmospheric deposition of metals, such as in the case of *Pleurozium schreberi* (Markert et al. 1994; Galsomies et al. 1999; Grodzinska and Szarek-Lukaszewska 2001; Galuszka 2006), *Scleropodium purum* (Markert et al. 1994; Fernández et al. 1999; Galsomies et al. 1999; Fernández and Carballeira 2001), *Hylocomium splendens* (Steinnes et al. 1994; Berg et al. 1995; Galsomies et al. 1999; Lucaciu et al. 2004; Galuszka 2006), *Plagiothecium denticulatum*, *Bryum argenteum* and *Sphagnum sp* (Gupta 1995), *Hypnum cupressiforme* (Fernández et al. 1999; Galsomies et al. 1999; Fernández and Carballeira 2001; Frontasyeva et al. 2004; Lucaciu et al. 2004; Pepi et al. 2006), *Thuidium tamariscinum* (Galsomies et al. 1999), *Brachytechium salebrosum* and *Brachytechium rutabulum* (Lucaciu et al. 2004), *Polytrichum formosum* (Markert and Weckert 2008) and *Sphagnum girgensohnii* (Aničić et al. 2009).

This work aims to evaluate the atmospheric deposition of the heavy metals Cr, Zn, Cd and Pb by using mosses that grow in the MATV, Mexico.

## Methods

### Sample collection

**Fig. 1:**
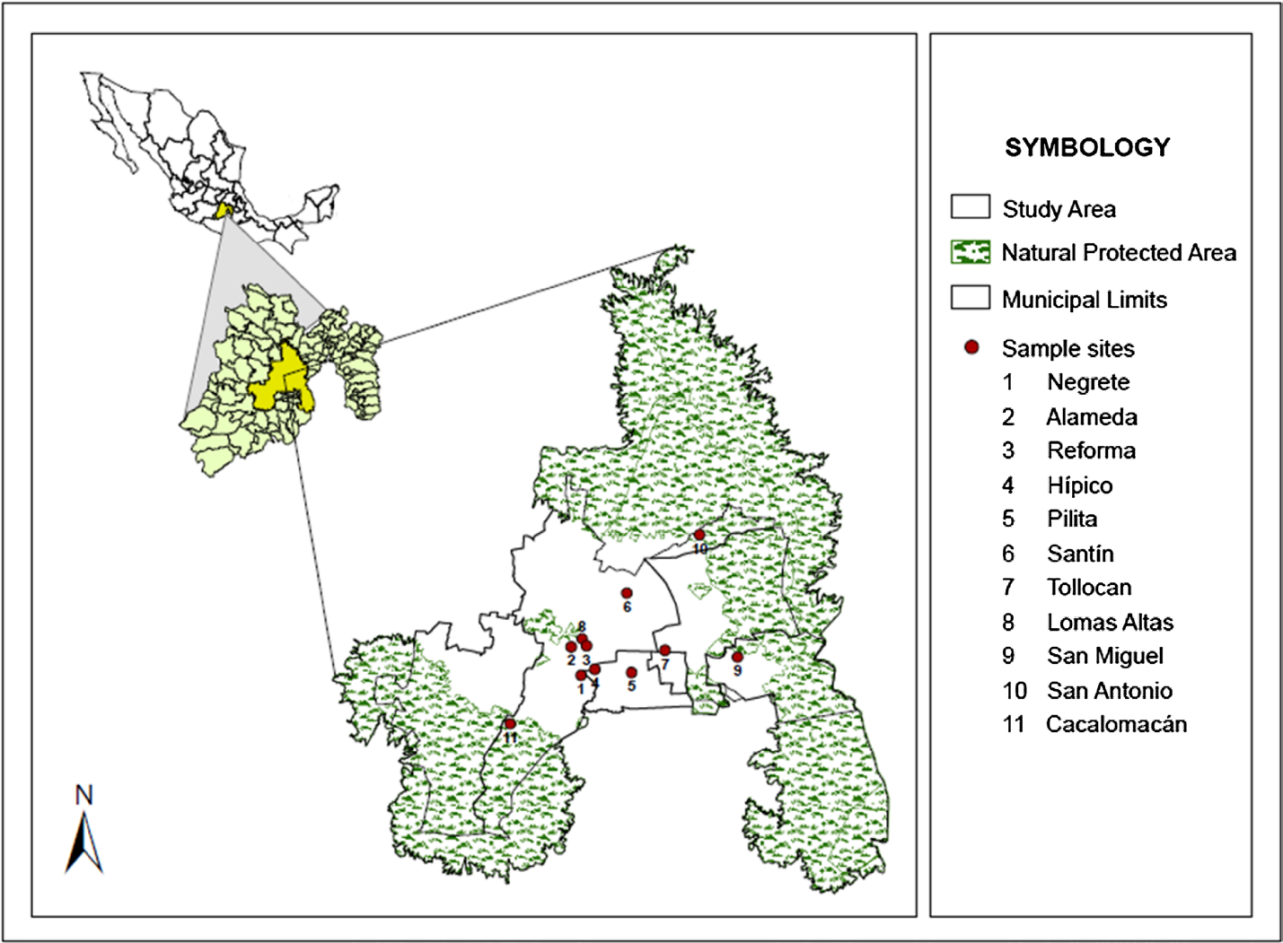
Moss sampling sites in the metropolitan area of the Toluca Valley

Eleven sampling sites distributed in the MATV were selected (Fig. 1). The sampling sites were selected taking into account the areas where moss was abundant, the sites with the highest percentage of tree cover and the location of the site with respect to air currents and sources of pollution in the area.

Epiphytes mosses were taken from eleven sites in natural areas and urban parks of the MATV. Two sampling campaigns were carried out in each site in a year period. The two sampling campaigns were conducted in August and November 2011 during the rainy and dry-cold season respectively. *Fabriona ciliaris* and *Leskea angustata*, the two most dominant species of epiphytic mosses found on the tree bark were collected. From ten trees with a height greater than one and a half meter, 10 g of epiphytic mosses were collected. Moss samples were removed from stems and branches by means of a plastic spatula and placed into polythene bags for transportation to the laboratory. Taxonomic identification and preservation techniques have been described by Zepeda-Gómez et al. (2014). In the laboratory, moss samples were washed with distilled water, dried at room temperature and ground in an agate mortar, then sieved through a 200-mesh sieve and finally homogenized in a mixer for 10 min.

### Sample preparation

0.3 g of each moss samples was digested by microwave oven (model Mars-X 907600 series XM3047 EMF) using a test method of CEM™ (Citrus Leaves-SRM 1572), adapted to the samples of mosses. The “Citrus Leaves-SRM 1572 Moss” (two-stage) method was used, firstly by adding 2 mL of ultrapure water, 5 mL HNO_3_ (Ultrex) and 1 mL HF (Ultrex) at the maximum temperature of 187 °C with a pressure of 240 psi, for 30 min with a holding time of 20 min; In the second stage (neutralization process), 15 mL of 4 % H_3_BO_3_ (analytical grade) was added to each vessel, and digestion at a temperature of 170 °C for 10 min was performed. After that, samples were decanted, filtered using paper Whatman 2 and placed in polyethylene containers and kept refrigerated until analysis. All standards and solutions were prepared with water type 1 (Resistivity 18.2 mΩ cm a 25 °C, Simplicity UV System, MILLIPORE).

### Absorption atomic spectrometry analysis

The Cr, Pb, Cd and Zn concentrations were determined by means of a Perkin Elmer 3110 Atomic Absorption Spectrophotometer and according with the Mexican standard NMX-AA-051-SCFI-2001 (SECOFI 2001). The detection of metals were performed at specific wavelengths for each element; Cr: 357.8 nm, Cd: 228.7 nm, Zn: 213.9 nm and Pb: 216.9 nm, using monoelemental hollow-cathode lamps. A five point calibration curve was generated by each one of the four elements. The linear correlation coefficient was higher than 0.997 for the four calibration curves (Macedo-Miranda et al. 2009).

The accuracy and reproducibility of the results were tested by means of control samples and duplicate samples analysis. Certified reference material (Lichen IAEA-336) was used to assess the accuracy of the analytical results. Certified reference material was prepared in the same conditions as the moss samples. Accuracy being measured as the percentage of recoveries after the acid digestion (ratio between values measured and certified in the reference material) was in the range of 90–110.

### Statistical methods

Average values, standard deviations and confidence limits were obtained by means of the Statgraphics program. In addition, the data was processed by means of SPSS Statistics v.17 software. A t test for independent samples and analysis of harmonized means ANOVA were performed to deduce significant differences in the concentration of heavy metals in the two species of mosses. Pearson correlation was used to confirm associations between metals in the mosses. To identify similarity in the heavy metal concentrations in mosses and grouping of clusters the different metal concentrations in mosses and the different sampling points, the factorial analysis was used. Also a principal component analysis was performed to show the different clusters using dendograms.

### Enrichment factors

In order to deduce anthropogenic or natural contributions of metals in mosses from the MATV, the enrichment factors (EF) were calculated. Iron was selected as conservative element (Salomons and Förstner 1984).

The average concentration of metals in the eleven sampling sites was used for the enrichment factor (Bargagli et al. 1995).

The next equation was used for the calculation of the enrichment factor (Lawson and Winchester 1979; Poissant et al. 1994).

$$EF = \frac{{\left( {\frac{C}{R}} \right)Moss}}{{\left( {\frac{C}{R}} \right)Soil}}$$

where C is the concentration of the element and R is the concentration of the reference element (in this case Fe) in the mosses and the soil.

Several criteria have been used in order to determine the levels of enrichment by metals in the mosses: enrichment factors ≤2 show that there is not enrichment of the element in the mosses and the element is coming from the soil (conservative); enrichment factors between 3 and 5 are considered slightly enriched; enrichment factors between 6 and 9 are considered moderately enriched; and enrichment factors ≥10 are considered highly enriched (Lantzy and Mackenzie 1979; Aničić et al. 2007; Dragovic and Mihailovic 2009; Zarazúa et al. 2013).

## Results and discussion

*Fabriona ciliaris* and *Leskea angustata* were the two main species of mosses collected in the sampling sites. Table 1 shows the dominant species in the sampling sites and sampling seasons.

**Table 1:** The main dominant species of epiphytic mosses by sampling site

Sampling site	Name	Sampling season/dominant specie	
		Dry-cold season	Rainy season
1	Negrete	*Leskea angustata*	*Leskea angustata*
2	Alameda	*Fabronia ciliaris*	*Fabronia ciliaris*
3	Reforma	*Fabronia ciliaris*	*Fabronia ciliaris*
4	Hípico	*Fabronia ciliaris*	*Fabronia ciliaris*
5	Pilita	*Fabronia ciliaris*	*Fabronia ciliaris*
6	Santín	*Leskea angustata*	*Leskea angustata*
7	Tollocan	*Fabronia ciliaris*	*Fabronia ciliaris*
8	Lomas Altas	*Leskea angustata*	*Fabronia ciliaris*
9	San Miguel	*Leskea angustata*	*Fabronia ciliaris*
10	San Antonio	*Leskea angustata*	*Leskea angustata*
11	Cacalomacan	*Leskea angustata*	*Leskea angustata*

Table 2 shows the concentration of Cr, Cd, Zn and Pb in the mosses from the MATV. In general, the average heavy metal concentrations (mg/kg) in the mosses are present in the next order: Zn > Pb > Cr > Cd, and the highest concentrations of the metals are in rainy season.

**Table 2:** Concentration of Cr, Cd, Zn and Pb in mosses from the MATV (mg/kg)

Sampling site	Cr		Cd		Zn		Pb	
	A	B	A	B	A	B	A	B
1	25.3 ± 0.1	29.4 ± 0.1	<0.1	0.6 ± 0.1	119.6 ± 16.1	85.9 ± 0.5	38.4 ± 0.7	43.9 ± 1.0
2	38.2 ± 0.8	40.9 ± 0.1	<0.1	0.6 ± 0.1	161.7 ± 1.6	145.4 ± 25.3	119.2 ± 0.9	86.6 ± 5.9
3	32 ± 0.1	47.0 ± 0.8	<0.1	0.2 ± 0.3	157.7 ± 0.1	153.8 ± 30.7	106.9 ± 0.1	140.2 ± 3.1
4	28.6 ± 0.1	25.8 ± 0.4	<0.1	0.3 ± 0.1	163.0 ± 0.1	10.8 ± 1.3	<0.5	43.9 ± 3.1
5	21.3 ± 0.1	27.2 ± 0.1	<0.1	0.3 ± 0.1	123.3 ± 0.1	107.4 ± 2.1	<0.5	27.4 ± 0.1
6	21.9 ± 0.1	21.6 ± 0.1	<0.1	7.3 ± 0.5	160.5 ± 0.1	200.2 ± 11.6	<0.5	34.0 ± 5.0
7	33.6 ± 0.1	45.6 ± 0.9	<0.1	3.5 ± 0.2	312.7 ± 0.1	428.5 ± 14.5	57.7 ± 0.1	93.4 ± 1.0
8	8.4 ± 0.1	19.7 ± 0.1	<0.1	<0.1	64.7 ± 0.1	88.9 ± 15.1	<0.5	23.4 ± 2.0
9	27.4 ± 0.6	45.3 ± 1.3	<0.1	0.9 ± 0.2	103.1 ± 0.3	163.8 ± 7.7	<0.5	69.4 ± 1.0
10	10.1 ± 0.1	17.8 ± 0.1	<0.1	0.2 ± 0.00	72.1 ± 0.1	85.0 ± 0.6	<0.5	37.5 ± 0.1
11	13.5 ± 0.1	14.9 ± 1.4	<0.1	0.6 ± 0.1	66.4 ± 0.1	66.6 ± 3.3	<0.5	13.5 ± 2.0
Average	23.7	30.5	<0.1	1.3	136.8	139.7	29.6	55.7

*A* dry-cold season

*B* rainy season

Cr concentrations, varies from 8.4 to 47 mg/kg with an average value of 23.7 mg/kg in the dry-cold season and 30.5 mg/kg in the rainy season. Cd concentrations, varies from <0.1 to 7.3 mg/kg with an average value of <0.1 mg/kg in the dry-cold season and 1.3 mg/kg in the rainy season. Zn concentrations, varies from 10.8 to 428.5 mg/kg with an average value of 136.8 mg/kg in the dry-cold season and 139.7 mg/kg in the rainy season. Pb concentrations, varies from <0.5 to 140.2 mg/kg with an average value of 29.6 mg/kg in the dry-cold season and 55.7 mg/kg in the rainy season.

Sites 2, 3, 6 and 7 present the highest heavy metal concentrations. These sites are associated with high vehicular traffic (Aničić et al. 2009), high emission of industrial pollutants (Aničić et al. 2007) and close to the influence area of the prevailing winds in the MATV that increases the heavy metal content in mosses (Wannaz, et al. 2006; Lijteroff et al. 2009; Zarazúa et al. 2013). After that, a lower heavy metal concentrations was found in sites 1, 4, 5, 9, and 10, with low vehicular traffic, low emission of industrial pollutants and outside the influence of the prevailing winds in the MAVT (SMA-GEM 2007b; Machado et al. 2008). Finally, the lowest heavy metal concentrations were found in the natural areas, (sites 8 and 11) where there is a large tree cover that reduce the influence of air pollution (Machado et al. 2007; Mejía-Cuero et al. 2015).

The higher concentrations of heavy metals in moss tissues in sampling sites 2, 3 and 7 are probably due to exposure of the mosses to high levels of air particulate matter with heavy metals (Fernández and Carballeira 2001; Onianwa 2001; Markert and Weckert 2008). In the rainy season, mosses increase their metabolism and biomass production, as well as metals are present in bioavailable form (soluble), for these reasons the concentration of heavy metals in the moss tissue of the second sampling campaign were the highest (Tessier and Boisvert 1999; Lucaciu et al. 2004; Aničić et al. 2009). In general, the concentration of heavy metal in mosses depends on the environmental conditions, the dry or rainy season, the ion exchange capacity of the mosses cell walls, the surface area of the moss's tissue and water content of the species (Grodzinska and Szarek-Lukaszewska 2001; Aničić et al. 2009).

The MATV is located in the influence area of the trade winds, the intensity of the trade winds is rather weak and stable in the dry-cold season with prevailing winds from the south and northbound in rainy season. The dominant winds are coming from the east and southeast with a direction of northwest and north. These conditions facilitate the aero-transportation of pollutants produced in the Toluca-Lerma and Ocoyoacac industrial areas and increasing the concentrations of heavy metals in some sampling sites (Wannaz et al. 2006). The Tollocan site is a good example, where increases in heavy metals concentration were observed. This site is located close to industrial area and it presents high vehicular traffic (Wannaz et al. 2006; Machado et al. 2007 and 2008; Caballero et al. 2014; Mejía-Cuero et al. 2015). The emissions inventory shows that 2 % of PM_10_ in the MATV corresponds to toxic heavy metals (Cr, Pb, Ni and Mn), which are produced by unsealed roads and soils without vegetation (SMA-GEM 2007b, 2012). Considering that, the presence of some heavy metals can be used as indicators of anthropogenic pollution, especially related to the industrial activity, traffic and vehicle emissions (Machado et al. 2007; Wannaz et al. 2012). It is possible to establish, that high levels of Cr, Cd, Zn and Pb in the Tollocan, Alameda, Reforma and Santín sites, are related principally with anthropogenic sources.

The global analysis through a simple t test for independent samples shows no statistical differences between the concentrations of heavy metals in the dry-cold and rainy season. However making a comparison between the heavy metal concentrations by species and sample season, it is possible to observe statistical differences in the concentrations between species for each period.

In dry-cold season a lower significant statistical concentration occurs of Cr (p = 0.007) and Zn (p = 0.036) in *Leskea angustata* compared with *Fabriona ciliaris* (p < 0.05), while there is not a significant statistical difference in the case of Pb concentration (p = 0.304) between the two species. In the rainy season, Cr (p = 0.039) has a significant statistical concentration lower in *Leskea angustata* than in *Fabriona ciliaris* (p < 0.05), and there is no significant differences in the case of Zn (p = 0.517) and Pb (p = 0.125) concentrations between the two moss species, even if the average concentration values are lower for *Leskea angustata*. In the case of Cd, it has a higher concentration in *Leskea angustata* compared with the concentration of this metal in *Fabriona ciliaris*. Based on the above, it is possible to establish that *Fabriona ciliaris* has a higher capacity to accumulate heavy metals in the MATV than *Leskea angustata* and for this reason it is a better biomonitor.

In the dry-cold season a significant statistical difference was observed in the higher concentrations of Cr (p = 0.04) in the urban area with regard to the natural areas (p < 0.05). Likewise, there were not significant differences in Cd, Zn (p = 0.080) and Pb (p = 0.306), even though the average values found in the natural areas were lower than urban areas. In the rainy season, there were not significant differences in the concentration of Cr (p = 0.195), Zn (p = 0.656), Cd (p = 0.669) and Pb (p = 0.407) in mosses between urban areas and natural areas, while the lowest average concentrations of metals appeared in the natural areas.

Pearson correlations results (Table 3) indicate a significant correlation (p < 0.05) between Cr and Zn and Cr and Pb in both sampling seasons and between Cd and Zn in the rainy season. The commonalities obtained in all cases show that the variables are well explained through the extracted components. The significant correlations (p < 0.05) between Cr, Zn, Pb and Cd indicate a common origin and/or shared contamination source for this metals.

**Table 3:** Significant Pearson correlations (p < 0.05)

Element	Cr	Cd	Zn	Pb
a. Dry-cold season				
Cr			0.72	0.730
Cd				
Zn	0.72			
Pb	0.73			
b. Rainy season				
Cr			0.58	0.89
Cd			0.55	
Zn	0.58	0.55		
Pb	0.89			

Results of principal component analysis (Table 4) show that in the dry season, two components explain the 94.14 % of the variance and three components explain the 100 % of the variance, while in the rainy season, two components explain the 91.51 % of the variance and three components explain the 97.61 % of the variance. In the dry season, the first component corresponds to the concentrations of Cr and Pb, the second component is represented by the concentrations of Zn and Cr and the third component by Cr concentrations. In the rainy season, the first component corresponds to the concentrations of Cr and Pb, the second component is represented by the concentrations of Cd and Zn and the third component by Zn and Cr concentrations.

**Table 4:** Total variance explained

Component	Dry-cold season			Rainy season		
	Initial eigenvalues			Initial eigenvalues		
	Total	Variance %	Cumulative %	Total	Variance %	Cumulative %
1	2.28	76.01	76.01	2.36	59.02	59.02
2	0.54	18.13	94.14	1.30	32.49	91.51
3	0.18	5.86	100	0.24	6.10	97.61
4				0.10	2.39	100

Principal component analysis

**Fig. 2:**
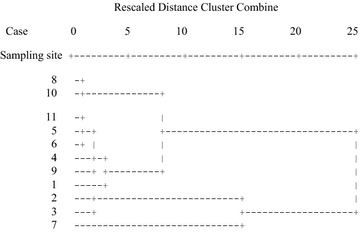
Dendrogram using average linkage (between groups) in dry season

**Fig. 3:**
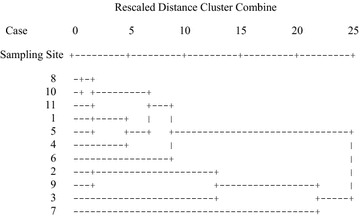
Dendrogram using average linkage (between groups) in rainy season

Figure 2 shows cluster analysis results in the dry season. The sites can be grouped in three clusters. The first cluster is formed by sites 1 (Negrete), 4 (Hipico), 5 (Pilita), 6 (Santin), 8 (Lomas Altas), 9 (San Miguel), 10 (San Antonio) and 11 (Cacalomacan), these sites have the lowest heavy metal concentrations in mosses. The second cluster is formed by sites 2 (Alameda) and 3 (Reforma), these sites have medium heavy metal concentrations in mosses; while the third cluster is formed by site 7 (Tollocan) and this site has the highest heavy metal concentrations in mosses. Figure 3 shows cluster analysis results in the rainy season. In the same way as in the dry season, the sites can be grouped in three clusters. The first cluster is formed by sites 1 (Negrete), 4 (Hipico), 5 (Pilita), 6 (Santin), 8 (Lomas Altas), 10 (San Antonio) and 11 (Cacalomacan), these sites have the lowest heavy metal concentrations in mosses. The second cluster is formed by sites 2 (Alameda), 3 (Reforma) and 9 (San Miguel), these sites have the medium heavy metal concentrations in mosses; while the third cluster is formed by site 7 (Tollocan) and this site has the highest heavy metal concentrations in mosses.

Results of enrichment factors (Tables 5, 6) show that Cr is conservative in both sampling seasons (EF ≤ 2), with a terrigenous origin, typical constituent of soils and aerosols (Zarazúa et al. 2013). Zn is moderately enriched in both sampling seasons (EF 6–9) which indicate that the sources were mainly pedological-soil or substrate contribution and anthropogenic activities (Zarazúa et al. 2013). Finally, Cd is highly enriched in the rainy season and Pb is highly enriched in both sampling seasons, with a predominantly anthropogenic origin (EF ≥ 10) (Ávila-Pérez et al. 2011; Zarazúa et al. 2013). Also, individual enrichment results show that in dry seasons, Zn in sampling sites 4 (Hipico), 7 (Tollocan) and Pb in sampling sites 1 (Negrete), 2 (Alameda), 3 (Reforma), 7 (Tollocan) are highly enriched (EF ≥ 10); in the case of the rainy season, Cd in sampling sites 1 (Negrete), 6 (Santin), 7 (Tollocan), 9 (San Miguel), 11 (Cacalomacan), Zn in sampling sites 7 (Tollocan), 8 (Lomas Altas) and Pb in all samplings sites are highly enriched (EF ≥ 10) (Ávila-Pérez et al. 2011; Zarazúa et al. 2013).

**Table 5:** Enrichment factors results in the dry season

Sampling site	Cr	Cd	Zn	Pb
1	1.8	ND	6.3	148
2	2.0	ND	6.4	344
3	2.6	ND	9.7	480
4	3.0	ND	12.8	ND
5	1.7	ND	7.3	ND
6	1.2	ND	6.6	ND
7	1.8	ND	12.4	168
8	1.6	ND	9.0	ND
9	2.3	ND	6.3	ND
10	1.5	ND	7.8	ND
11	2.3	ND	8.3	ND
Average	2.0	ND	8.4	104

*ND* not determined because value was <limit of detection

**Table 6:** Enrichment factors results in the rainy season

Sampling site	Cr	Cd	Zn	Pb
1	2.5	13.8	4.4	165
2	2.4	9.5	5.1	224
3	3.5	3.8	6.7	450
4	2.2	8.4	0.7	204
5	2.1	6.0	4.8	91
6	1.7	165	9.2	115
7	3.2	70	17.6	282
8	4.0	ND	10.7	206
9	3.4	19.2	7.4	320
10	1.9	5.5	5.3	171
11	2.2	28	5.9	87
Average	2.6	30	7.1	210

*ND* not determined because value was <limit of detection

According to the results, mosses are able to concentrate heavy metals, which is a great advantage for urban areas where these species grow naturally and can be used as biomonitors to assess the air pollution level. Trace elements are deposited on the surface of mosses, as dry particulate matter or dissolved material. The heavy metals are retained by adsorption, physico-chemical processes such as ion exchange or passive-active intracellular uptake and it has been suggested that this latter mechanism is the dominant process in the bryophytes (Onianwa 2001). Bioaccumulation process is regulated by the chemical form of metals, principally ionic forms and by the affinity between the chemical form of metals and the biochemical structures in mosses (Klos et al. 2011).

Mosses have been used in Northern Europe since the end of the 1960s for tracking the pollution as an indirect measure of atmospheric deposition of heavy metals (Fernández and Carballeira 2001). Epiphytic mosses have proved to be excellent bioindicators for atmospheric pollution (Dragovic and Mihailovic 2009). So, the concentration of elements in the moss's tissues reflects the atmospheric heavy metal deposition. Finally, this technique is so useful for monitoring the air particulate matter quality and also an economical environmental tool (Aničić et al. 2009; Ares et al. 2015).

Given that the results are based on four heavy metals and two moss species, the study must be expanded to other metals, moss species and interspecies comparison. Since, heavy metal concentrations in moss's tissues have been used to develop air pollution maps in others countries, these results should be used to produce pollution maps for the MATV.

## Conclusions

The epiphytes mosses that were collected in the urban areas showed the highest concentrations of Cr, Cd, Zn and Pb and a seasonal variation. The lowest concentrations of heavy metals present in the moss's tissues correspond to unpolluted areas. The average heavy metal concentrations in the mosses are present in the next order: Zn > Pb > Cr > Cd, and the highest concentrations of heavy metals are presents in urban sampling sites 2 (Alameda), 3 (Reforma), 6 (Santin) and 7 (Tollocan) in the rainy season. The highest heavy metal concentrations in urban sites are related to high vehicular traffic and high emission of industrial pollutants. *Fabriona ciliaris* showed a higher capacity to accumulate heavy metals in the MATV than *Leskea angustata* and for this reason it is a better biomonitor for environmental pollution. Cr is conservative in both sampling seasons (EF ≤ 2). Zn is moderately enriched in both sampling seasons (EF 6–9). Cd and Pb are highly enriched in the rainy season and in both sampling seasons respectively (EF ≥ 10). The use of biomonitors in studies of environmental pollution has several advantages compared with the use of air filters or deposition samplers. The benefits are related to the simplicity of the sampling, the accumulation level of heavy metals and the use of cheaper equipment, in such a way that the biomonitoring by means of the mosses species *Leskea angustata* and *Fabronia ciliaris* is a viable procedure for the evaluation of environmental pollution in the MATV.
